# ADRD CAREGIVERS’ ROLE PERCEPTION AND MENTAL HEALTH AFTER LTC-PLACEMENT OF FAMILY MEMBERS

**DOI:** 10.1093/geroni/igad104.1229

**Published:** 2023-12-21

**Authors:** Hyejin Kim, Olimpia Paun, Jessica Bishop-Royse, Ben Inventor, Masako Mayahara, Sarah Ailey, Louis Fogg

**Affiliations:** Rush University College of Nursing, Chicago, Illinois, United States; Rush University, Chicago, Illinois, United States; Rush University, Chicago, Illinois, United States; Rush University, Chicago, Illinois, United States; Washington University in St. Louis, St. Louis, Missouri, United States; Rush University, Chicago, Illinois, United States; University of Illinois at Chicago, Chicago, Illinois, United States

## Abstract

The Chronic Grief Management Intervention-Video (CGMI-V) clinical trial tested the effects of an 8-week live online intervention for caregivers who placed their family members with ADRD in LTC on caregivers’ mental health outcomes (grief, depression and anxiety symptoms, positive states of mind). This cross-sectional study used baseline data (n=95) from the CGMI-V. We performed hierarchical linear regression to examine the main effects of caregiving role perceptions on caregivers’ levels of grief, depression, anxiety symptoms, and positive states of mind, controlling for potential covariates. At baseline, perceived caregiver role captivity was the most significant factor of outcome variables, followed by feelings of guilt and loss. Increased feelings of role captivity were linked to sum scores of grief as measured with the Marwit-Meuser Caregiver Grief Inventory. These findings indicate that increased feelings of role captivity were associated with increased feelings of loss and loss of interpersonal connections. Higher levels of perceived role captivity were associated with more depressive and anxiety symptoms. Caregivers with lower levels of perceived role captivity had higher levels of positive states of mind. The findings confirmed the need for interventions targeting caregivers’ feelings of role captivity, loss, and guilt related to caregiving obligations post-LTC placement of family members with ADRD to reduce caregivers’ grief, depression, and anxiety and to increase their positive states of mind. These baseline findings are preliminary. Future presentations will report the effects of the CGMI-V intervention on caregivers’ grief and mental health, including weeks 8 and 24 follow-up data.

